# Microbial Safety of Milk Production and Fermented Dairy Products in Africa

**DOI:** 10.3390/microorganisms8050752

**Published:** 2020-05-17

**Authors:** James Owusu-Kwarteng, Fortune Akabanda, Dominic Agyei, Lene Jespersen

**Affiliations:** 1 Department of Food Science and Technology, University of Energy and Natural Resources, BS-0061-2164 Sunyani, Ghana; 2 Department of Applied Biology, University for Development Studies, P.O. Box 24, NT-0272-1946 Tamale, Ghana; fakabanda@gmail.com; 3 Department of Food Science, University of Otago, Dunedin 9054, New Zealand; Dominic.Agyei@otago.ac.nz; 4 Department of Food Science, Food Microbiology, University of Copenhagen, Rolighedsvej 26, DK 1958 Frederiksberg Copenhagen, Denmark; lj@food.ku.dk

**Keywords:** pathogens, pasteurization, raw milk, fermentation, microbial hazards, HACCP, dairy

## Abstract

In Africa, milk production, processing and consumption are integral part of traditional food supply, with dairy products being a staple component of recommended healthy diets. This review provides an overview of the microbial safety characteristics of milk production and fermented dairy products in Africa. The object is to highlight the main microbial food safety hazards in the dairy chain and to propose appropriate preventive and control measures. Pathogens of public health concern including *Mycobacterium bovis*, *Brucella abortus* and *Coxiella burnettii*, which have largely been eradicated in many developed nations, still persist in the dairy chain in Africa. Factors such as the natural antimicrobial systems in milk and traditional processing technologies, including fermentation, heating and use of antimicrobial additives, that can potentially contribute to microbial safety of milk and dairy products in Africa will be discussed. Practical approaches to controlling safety hazards in the dairy chain in Africa have been proposed. Governmental regulatory bodies need to set the necessary national and regional safety standards, perform inspections and put measures in place to ensure that the standards are met, including strong enforcement programs within smallholder dairy chains. Dairy chain actors would require upgraded knowledge and training in preventive approaches such as good agricultural practices (GAP), hazard analysis and critical control points (HACCP) design and implementation and good hygienic practices (GHPs). Food safety education programs should be incorporated into school curricula, beginning at the basic school levels, to improve food safety cognition among students and promote life-long safe food handling behaviour.

## 1. Introduction

Historically, milk and dairy products have been significant components in the diets of Africans and continue to play an important and increasing role in the diets of the growing population of both rural and urban communities [1,2,3]. Generally, milk and dairy products are rich in nutrients, delivering high quality proteins, micronutrients, vitamins and energy-containing fats [4,5]. Milk, thus, provides an ideal environment for the growth of wide variety of food-borne microorganisms and zoonotic agents [6]. The microbiological quality of milk, at the point of milking from a healthy animal, is theoretically expected to be safe for human consumption. However, once it is secreted from the udder, milk can easily be contaminated by spoilage microorganisms and food-borne pathogens from various sources including animal faeces, soil, air, feed, water, equipment, animal hides and people. Thus, the prevalence of pathogenic and spoilage microorganisms in milk and dairy products is influenced by a high number of factors and their combinations. These factors may include health status of the dairy herd, hygiene level in the dairy farm environment, milking and prestorage conditions, available storage facilities and technologies, farm management practices, geographic location and season [7,8]. In addition to microbial hazards, milk and dairy products can also contain chemical hazards and contaminants mainly introduced through the environment, animal feedstuffs, animal husbandry and industry practices. Thus, safety and production are intrinsically linked in the dairy food chain; from production through handling and processing to consumption. Therefore, in order to minimize the food safety risks associated with milk and dairy products, there is the need for a continuous system of preventive measures beginning with safety of animal feed, through good farming practices and on-farm controls, to good manufacturing and hygiene practices, consumers safety awareness, and proper application of food safety management systems throughout the dairy chain [9].

Food-safety risks associated with raw milk and dairy products consumption vary considerably between developed and developing countries. While the dairy sector in developed nations is largely industrialized, characterized by routine application of pasteurization technologies, the dairy sector in developing countries is dominated by many smallholder dairy farmers and processors [9]. In most countries in Africa, the informal sector which handles most of the milk and dairy products is characterized by unpasteurized milk sold through small-scale channels that lack a cold chain and has little or no regulatory control [10,11]. This review, therefore, provides a comprehensive overview of the microbial food safety issues associated with raw milk production and traditional dairy products in Africa. Additionally, the paper discusses the contribution of factors such as natural antimicrobials components in milk and traditional milk processing methods to the safety of raw milk and dairy products in Africa. Finally, practical steps aimed at ensuring safety in the African dairy chain are proposed.

## 2. Food Safety Hazards in the Dairy Chain

Food-safety hazard generally refers to any biological, chemical or physical agent in a food, or condition of food with the potential to cause adverse health consequences for consumers [12]. Such hazards may be introduced into the dairy chain at any time during primary production, milking, formulation and processing, packaging and labelling, transportation, storage, preparation, and serving. Major food safety hazards associated with raw milk production and dairy products may be put into three categories: biological, chemical and physical, as shown in Table 1 [13]. From raw milk production through processing to the consumer, milk is exposed to several hazards, which ultimately influence the safety and quality of the final product. Some of these hazards may stem from animal husbandry practices, through their feeding, milking and processing.

**Figure 1:**
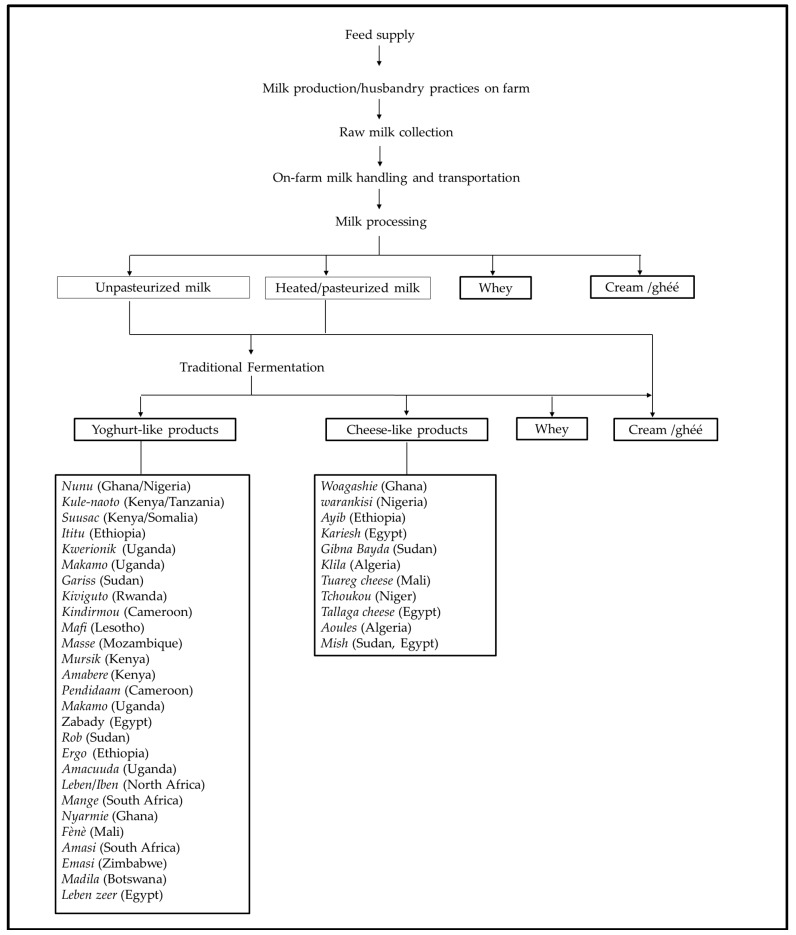
Overview of various stages involved in common African traditional dairy chains.

Generally, the traditional production of raw milk and dairy products in Africa follows few common stages beginning with animal feed supply, followed by the production of raw milk which may be sold directly to consumers without processing, or further processed into various traditional products by small scale processors. Typically, along the dairy chain in Africa, milk may be consumed as nonpasteurized milk, heated milk or processed into various fermented yoghurt-like and cheese-like products, as shown in Figure 1.

## 3. Risk Factors for Microbiological Hazards in Dairy Production and Processing in Africa

From a healthy animal, raw milk is expected to harbour no pathogens at the point of collection. However, this is seldom the case. Generally, pathogenic microorganisms can contaminate raw milk in two ways. First, endogenous contamination occurs when milk is contaminated by a direct transfer of pathogens from the blood (systemic infection) of an infected animal into the milk, or via an infection in the udder. The second means by which fresh milk can be contaminated, known as exogenous contamination, occurs where milk is contaminated during or after collection by animal faeces, the exterior of the udder and teats, the skin, and other environmental sources [14]. Table 2 summarizes important risk factors and their implications for milk safety.

During the primary production stage, pathogens can contaminate milk through various routes. Animal feed and drinking water often serve as sources of microbial contamination. Predominant among the dairy production systems in Africa are the rural smallholder dairies in which farm animals are fed on grass, crop residues and cultivated fodder, or they roam the land in search of grazing grounds and water. Consequently, the microbiological quality of feed and water for dairy animals are not routinely assessed under these systems, and therefore there is a high risk for the ingestion of contaminated feed and water by dairy animals in Africa. Upon ingestion of contaminated water or feed, surviving (spore-formers) pathogens can be expelled into the farm environment and subsequently attach to teats and udder of dairy animals. Apart from contaminating the external surfaces of udder and teats, several potential pathogens including the genera *Staphylococcus*, *Streptococcus*, *Bacillus*, *Micrococcus*, and *Corynebacterium* can colonize the mammary glands of dairy animals even without any disease symptoms [15].

Mastitis, an inflammation of the mammary glands and udder tissues, is caused by a large variety of common bacteria, fungi, mycoplasmas and algae [16] infecting the mammary glands of dairy animals. Mastitis adversely affects animal health, milk quality, consumer safety, and can lead to great economic losses for milk production [17,18,19]. In sub-Sahara Africa, both subclinical mastitis (SCM) and clinical mastitis (CM) among dairy cows have been reported with prevalence rates in the range of 16.1%–90.3% and 4.8%–25.5%, respectively [20,21,22]. A recent report indicates that the prevalence of subclinical mastitis among dairy cows in some districts in Rwanda is 50.4% and the milk collected from positive mastitis cows were found to harbour coagulase negative Staphylococci (51.5%), *Staphylococcus aureus* (20.6%), *Streptococcus* species (10.3%), *Bacillus* species (10.3%), *Streptococcus agalactiae* (5.8%), and *Escherichia coli* (1.5%) [23]. Mastitis among dairy herds is a major constraint, and the disease has been identified as a primary cause of poor-quality and compromises the safety of raw milk in Ethiopia [24]. Generally, milk can easily become contaminated before it is secreted from the udder owing to mastitis. Although microbial contamination of milk during milking is difficult to completely avoid [25], it is of utmost importance to maintain a very high level of hygiene in dairy farming practices and proper cleaning of teats during milking for good udder health and optimum milk quality, and to ensure safety [26,27].

Once milk is secreted from the udder, it can be contaminated from several sources including air, faeces, bedding material, soil, feed, water, equipment, animal hides and people. A critical factor affecting milk safety is milking hygiene. Adequate milking hygiene potentially reduces the contaminating microorganisms and prevents them from inhabiting the immediate environment or skin of the animals, hands personnel and milking equipment, thereby minimizing their spread during milking [28]. High prevalence of pathogens in raw milk and occurrence of mastitis have previously been recorded in farms that practiced poor milking hygiene in Africa [22,29,30,31]. The dairy farm environment can be a reservoir of foodborne pathogens and serve as a major source of microbial contamination of raw milk due to direct contact with the milk. The use unsterilized collection vessels (containers) and other practices such as milking with unsanitized bare hands and allowing calves to feed without cleaning the teats of udders, expose milk to microbial contamination. In most small-scale milk production farms in Africa, there are no strict implementations of procedures for cleaning and disinfection of materials used during production processes, from milking to the sales of final products. While most foodborne pathogens such as *E. coli*, *Salmonella* spp. and *Campylobacter* spp. inhabit the ruminant intestinal tract, others including *Listeria* spp. and *Bacillus* spp. are widespread in nature and live in soils and plant environments. Thus, these environmental microorganisms can contaminate the milk by direct contact or through milking equipment and personnel on the farm if good hygiene management practices are not followed. Additionally, intentional adulterated of raw milk with contaminated water has been reported [32], a practice that potentially serves as another major source of pathogenic microorganisms in raw milk.

Storage and transportation of raw milk immediately after milking through to point of sale or processing are critical for safety and quality. In order to prevent proliferation of pathogens that contaminate freshly collected milk, there should be strict time and temperature controls between the milking and the processing of dairy products. Ideally, raw milk should be immediately cooled to below 4 °C to prevent microbial growth and ensure high-quality, safe milk for processing and consumption. This is in practise not possible for most small-scale producers in Africa, and therefore pasteurisation and sterilization of the milk are strongly recommended. This is unfortunately often not the case. Modern cooling facilities including mechanical refrigeration or cooling tanks are not available to the many small-scale dairy producers for reasons such as high initial investment and running costs and technical problems, including the lack or unreliable supply of electricity. Additionally, the majority of raw milk producers in Africa are mostly located in remote rural areas with poor road networks making it difficult to transport milk to urban markets and small-scale processing units. Subsequently, raw milk is often transported from the farm to small-scale processing units in urban market centres by bicycles, motorcycles, animals (donkeys), or by foot. The usual high ambient temperatures, often reaching 35–42 °C in most parts of sub-Sahara Africa, highlight the problem by accelerating the growth of spoilage and pathogenic microorganisms during transportation of raw milk.

The majority of milk produced in Africa is processed into a variety of traditional milk products by small-scale processing units or processors. The final products including spontaneously fermented yoghurt-like milk, traditional cheeses and butter, are produced with slight variations in processing methods depending on country or local region, which is affected by local tastes, dietary habits or culinary traditions [33,34]. The production of African traditional dairy products is based on recipes handed down from one generation to another, and processors often do not have access to formal training but learn by seeing, hearing and practicing [34]. Small-scale processors of traditional milk products often lack pasteurization, storage and packaging facilities and do not adhere to good manufacturing and hygiene practices (GMP/GHPs), including the implementation of starter culture procedures for milk fermentation. The processing of milk into yoghurts and other fermented products in most parts of Africa relies on spontaneous fermentation or back-slopping where a part of a previous batch of a fermented product is used to inoculate the new batch [34,35,36]. Consequently, traditional African fermented milk products may be susceptible to contamination with human pathogens of public health concerns due to the lack of proper control measures and adherence to good manufacturing practices (GMP) in the traditional fermentation processes.

## 4. Pathogens Occurring in Raw Milk and Dairy Products in Africa

**Figure 2:**
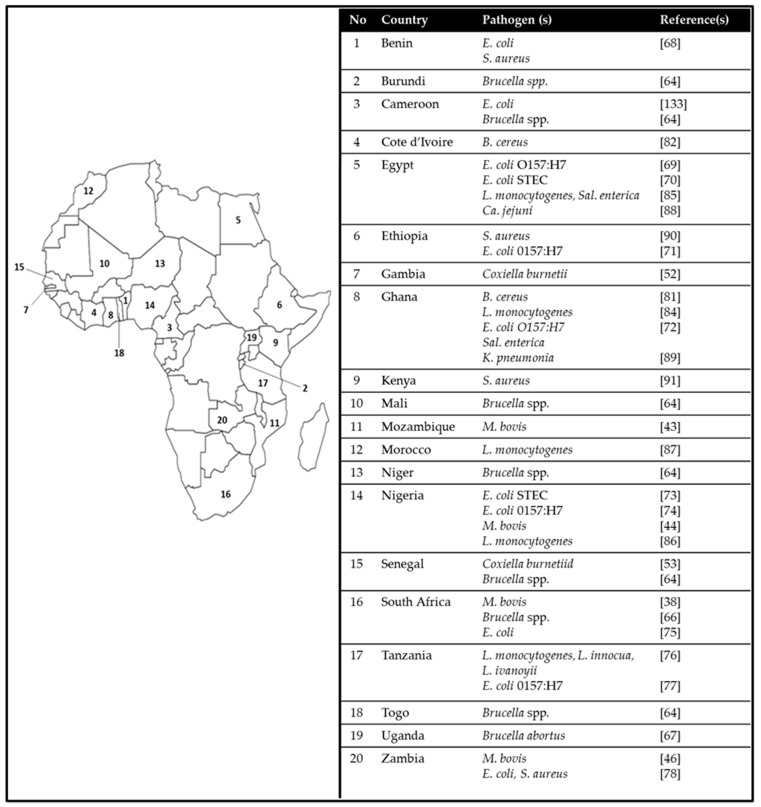
Pathogenic microorganisms occurring in milk and dairy products in some African countries. *E.*: *Escherichia*; *S.*: *Staphylococcus*; *B.*: *Bacillus*; *L.*: *Listeria*; *Sal.*: *Salmonella*; *Ca.*: *Campylobacter*; *K.*: *Klebsiella*; *M.*: *Mycobacterium*.

The major pathogens of concern in milk and dairy products have traditionally included *Mycobacterium bovis*, *Brucella abortus* and *Coxiella burnettii*, which are the causative agents of bovine tuberculosis and a form of human tuberculosis, brucellosis and Q fever, respectively. Unfortunately, while these pathogens and their diseases have been reported to be largely eradicated in many developed countries, they still persist or are re-emerging in some countries in Africa [37,38,39,40,41]. An overview of common pathogenic microorganisms occurring in milk and dairy products in some African countries is presented in Figure 2.

*Mycobacterium bovis*, the causative agent for bovine tuberculosis, has been detected in milk and dairy products in different African countries including South Africa [38,42], Mozambique [43], Nigeria [44], Tunisia [45] and Zambia [46]. Thus, the consumption of unpasteurized raw milk and dairy products continue to be a major risk for exposure to *M. bovis* in Africa. While bovine tuberculosis is known to be widespread in Africa, the limited or lack of sufficient data to expose the true epidemiological picture and burden of the disease in many African countries is a major concern, particularly when the burden of bovine tuberculosis might be considerably underestimated in humans [47,48,49].

*Coxiella burnetii*, an obligatory intracellular Gram-negative bacterium belonging to the family of Coxiellaceae is the causative organism of Q fever, a zoonosis of almost worldwide distribution except in New Zealand [40,50,51]. The most common reservoirs for *C. burnetii* include cattle, sheep and goats, and are considered the main sources of human infection [50]. Thus, consumption of non-pasteurized milk and their products in Africa may be a significant source of human contamination with *C. burnetii*, as this pathogen has been detected in up to 63% of cattle milk samples in Nigeria [40]. Furthermore, *C. burnetii* has been detected in milk samples in Gambia [52] and Senegal [53]. The presence of *C. burnetii* in milk samples raises concern on the role of milk as a source for human infection, particularly in regions where unpasteurized milk is consumed [54]. While Q fever is usually not considered a tropical disease, *C. burnetii* was found as the etiological agent in 5% of severe pneumonia cases in Tanzania [55]. Additionally, a study of a cohort of severely ill febrile patients in Tanzania revealed 26.2% zoonoses, among which 30% were reportedly Q fever [56]. Furthermore, *C. burnetii* accounts for about 1 to 3% of infective endocarditis in Tunisia and Algeria while Q fever accounts for about 5% of acute febrile illnesses in Burkina Faso [40]. About 9% of community-acquired pneumonia among patients aged above 15 years in Cameroon tested positive for *C. burnetii* [40], with *C. burnetii* being the third most frequently isolated agent of pneumonia, after *Streptococcus pneumoniae* and *Mycoplasma pneumoniae* in Cameroon [57]. 

Species of Brucella including *Brucella abortus*, *B. melitensis*, *B. suis* and *B. canis* are all capable of producing brucellosis in humans, with the disease being considered to represent one of the highest public health burdens of any zoonosis globally [58,59,60]. Ruminants are the primary hosts for *B. abortus* and *B. melitensis*, and humans become infected by consuming raw milk and dairy products, by direct contact with aborted foetuses, afterbirth and parturition fluids and during slaughter practices [61,62,63]. Although there is scanty prevalence data on brucellosis in Africa, it is suspected that the disease may be endemic in the region due to the high level of infection among dairy herds in different parts of the region [64]. Banfo et al. [65] estimated that up to 30% of milk and dairy products at selling points in Bamako, Mali were contaminated with Brucella. More recently, *Brucella* spp., particularly *B. abortus*, have been reported in milk and dairy products with high prevalence in some African countries such including South Africa [66], Uganda [67], Togo, Mali, Burundi, Cameroon, Senegal and Niger [64]. These reports indicate that brucellosis or the causative microorganisms are widespread among dairy supply chains of Africa, and this presents a serious public health threat to local populations, particularly consumers of raw milk and traditional dairy products, as well as dairy farm workers.

Other pathogens of significant safety concern in the African dairy chain are toxigenic strains of *Escherichia coli*, *Bacillus cereus* and *Listeria monocytogenes*. Strains of toxigenic *E. coli* have been reported in raw milk from different African countries such as Benin [68], Egypt [69,70], Ethiopia [71], Ghana [72], Nigeria [73,74], South Africa [75], Tanzania [76,77] and Zambia [78]. Shiga toxin producing *Escherichia coli* (STEC), has emerged as a group of highly pathogenic *E. coli* strains characterized by the production of one or more Shiga toxins. Similarly, *B. cereus* are of particular concern in food safety and public health due of their capacity to cause disease in humans through the production of various forms of enterotoxins and emetic toxins [79,80]. Strains of *B. cereus* possessing various forms of virulent factors have been detected in raw milk and traditional dairy products in Ghana [81] and Cote d’Ivoire [82]. *Listeria monocytogenes*, among other human pathogens, is considered a major microbiological and public threat associated with consumption of raw milk. *L. monocytogenes* has traditionally been a major public health issue in temperate regions including Europe and the US, particularly, due to their ability to grow at low temperature environments [83]. However, they have recently been isolated from different animal and milk products across Africa. The prevalence and characteristics of *L. monocytogenes* in raw milk and traditional dairy products in Ghana has been reported [84]. *L. monocytogenes* has also been recently reported to be prevalent in milk from other African countries including Egypt [85], Nigeria [86], Morocco [87] and Tanzania [76]. Other pathogens that have been detected in milk and milk products in Africa include *Campylobacter jejuni* [88], *K. pneumonia* [89] and *S. aureus* [90,91].

## 5. Factors that Potentially Contribute to the Safety of Milk and Dairy Products in Africa

### 5.1. Natural Antimicrobial Systems in Milk

Generally, raw milk contains natural antimicrobial peptides and enzymes including lactoferrin, lactoperoxidase, lysozyme and N-acetyl-β-D-glucosaminidase, which may enhance the microbial safety of raw milk. These natural inhibitory systems in milk may prevent a significant increase in microbial loads within the first 3–4 h after harvesting milk at ambient temperatures [92]. These natural antimicrobials inhibit postharvest bacterial growth in the raw milk, thereby protecting consumers of raw milk against pathogenic microorganisms.

Lactoferrin is a glycoprotein with two binding sites for iron, and is found predominantly in colostrum, milk and other mammalian body secretions such as saliva, vaginal and seminal fluids and tears [93,94,95]. Lactoferrins present in bovine milk vary in amino acid composition but generally constitute about 3% of the total milk protein [96]. In the colostrum, lactoferrin concentration is higher in both human and cow milk but decreases during the lactation period to insignificant levels. Several studies have reported on the physiological and antimicrobial functions of lactoferrins [94,97,98,99]. The antimicrobial effects of lactoferrin can be direct through bacteriostatic and bactericidal activity or indirect through activation of a complex series of reactions that lead to a protective immune response following microbial infections [100,101]. In vitro experiments and other studies have proven that one mechanism underlying the antimicrobial properties of lactoferrin is due to the iron-chelating property, which deprives microbes of iron, an essential nutrient necessary for growth. Thus, the bacteriostatic effect of lactoferrin is lost upon saturation of lactoferrin with iron [102,103,104]. Sometimes, however, the iron-chelating property of lactoferrin results in the death of some bacteria but encourages the growth of other bacteria with low iron requirement for growth [101,105]. Another mechanism for the antimicrobial property of lactoferrin is the direct interaction of intact or partially hydrolysed lactoferrin with lipopolysaccharide of the microbial cell, which may disrupt the cell wall integrity through dispersion of lipopolysaccharides, resulting in cell lysis [93,106,107]. Lactoferrin was found to inhibit the growth of *E*. *coli* and *P*. *aeruginosa* at concentrations of 0.67mg/mL, 1.67mg/mL and 2.67mg/mL but not *S*. *aureus*, *K*. *pneumonia* and coagulase-negative staphylococci isolated from a mastitic bovine udder [94]. Lactoferrin indirectly plays a role in cellular defence against microbial invasion by influencing the production of lymphocytes and macrophage activities [107]. Generally, the antimicrobial activities of lactoferrins are not significantly affected by commercial pasteurisation. However, treatments above temperatures used for pasteurisation can lead to their inactivation [108,109,110]. Thus, lactoferrins may contribute to the safety of raw or pasteurized milk and can complement—but cannot substitute—good hygienic practices in milk production and processing.

Lactoperoxidase, also known as milk peroxidase, is one of the heat-stable enzymes which is initially present at low concentrations in cow colostrum but increases after delivery. Like peroxidase found in tears, saliva, intestinal as well as nasal and bronchial, lactoperoxidase plays a protective role in the mammary gland, preventing microbial invasion [111]. In milk, lactoperoxidase alone has no significant antimicrobial activity. However, in the presence of hydrogen peroxide, lactoperoxidase causes the oxidation of thiocyanate ions into hypothiocyanous acid, which dissociates quickly in raw milk to hypothiocyanite ions. These hypothiocyanite ions are transient but have potent bacteriostatic effect against most mesophilic bacteria present in raw milk when oxidized by free sulphydryl groups. This occurs due to inactivation of important metabolic enzymes in bacteria consequently shutting down the cell metabolism and hence cell growth. This natural system is known as the lactoperoxidase system (LP-system). Lactoperoxidase is also reported to possess antiviral activity [112,113]. Activation of the LP-system helps to slow down microbial growth while transporting raw milk in remote areas having real difficulties with the application and maintenance of a cold chain system by smallholder dairy producers as pertains in many parts of Africa. The FAO/WHO in exploring ways to increase milk availability in Mali used thiocyanate and hydrogen peroxide to reactivate lactoperoxidase in raw milk. This treatment inhibited bacterial growth at ambient temperature, enabling milk to be transported to collection centres without spoilage losses and safety problems [114]. However, other safety concerns regarding the use of such system to preserve milk exist. Consequently, the FAO/WHO recommended that milk treated with the lactoperoxidase system should not be traded in the international market, that the use of the lactoperoxidase system should not be a substitute for pasteurization where possible, and that proper refrigeration and good hygiene should be practiced for securing the safety and quality of milk [115]. 

Lysozyme is another enzyme in milk, which acts synergistically with other antimicrobials to enhance the safety of raw milk. Lysozyme occurs in low concentrations in bovine raw milk but these levels are not reduced during pasteurisation because the enzyme is heat stable [116,117,118]. However, cows infected with mastitis have significantly higher concentrations of lysozyme in their raw milk compared to noninfected cows. The antibacterial activity of lysozyme is effective when working together with lactoferrin or immunoglobin A. For instance, the growth of *E. coli* was inhibited by the action of lysozyme and immunoglobin A [119]. Lysozyme causes lysis of some *Salmonella* spp. in association with ascorbate and peroxide, both of which are present in low concentrations in milk. However, lysozyme alone as a biopreservative at concentrations up to 5 mg/mL, was not successful at inhibiting the growth and biofilm formation of *S. aureus* isolated from raw milk and cheese [120].

### 5.2. Traditional Milk Processing Methods

Throughout Africa, raw milk is processed into various traditional products including yoghurts and cheeses [33,34,121,122,123]. The main risks associated with the consumption of raw milk or its products are mainly of a microbiological nature. It has been shown that consumption of raw unpasteurized milk and its products pose realistic health threats due to possible contamination with human pathogens [14]. Therefore, it is prudent that various precautionary measures are put in place during production, handling and processing of milk to ensure safety of consumers.

Traditional processing of milk in Africa employs various unit operations or techniques which may enhance the safety of milk. These processing techniques or operations include thermal treatments, fermentation, or the use of other antimicrobials additives during processing. In the following sections, we explore the potential roles of these traditional processing techniques in enhancing the safety of milk products in Africa.

#### 5.2.1. Heat Treatment

The microbial risks associated with the consumption of raw milk can be significantly reduced or completely eliminated by heat treatment. To prevent over-growth of surviving pathogens that contaminate the milk during harvest or incidental recontaminations during processing, pasteurization and temperature control (rapid cooling, chilled storage) are critical control points for foodborne pathogens associated with milk [124]. Previous data provide convincing evidence that adequate pasteurization of milk enhances safety and improves public health [125,126]. Depending on the time–temperature combinations applied, heat treatment of milk can be categorized as thermization (57 °C –68 °C for 15–20 s), pasteurization (60 °C–65 °C for 30 m or 71–74 °C for 15–40 s) or sterilization (110 °C –120 °C for 10–20 min) which includes ultra-high temperature (UHT; 135–140 °C for 6–10 s for indirect and 140–150 °C for 2–4 s for direct UHT) and innovative steam injection (ISI; 150–200 °C for < 0.1 s) treatments. Each of these heat treatments of milk aim at different microbial targets and result in different shelf-life of the treated milk [125]. For example, thermization generally leads to only a 3–4 log reduction in the counts of the vegetative commensal microorganisms such as *Aeromonas* spp., coliform bacteria, *Enterobacter* spp., *Micrococcus* spp. and *Pseudomonas* spp., of milk, but does not completely inactivate all vegetative pathogens. On the other hand, pasteurization can eliminate all vegetative microorganisms, including vegetative human pathogenic cells of *E. coli*, *Salmonella* spp., *L. monocytogenes*, *Yersinia enterocolitica*, *Campylobacter jejuni*, enterotoxin producing *S. aureus* and *Clostridium botulinum* which may be present in raw milk [125]. However, pasteurization does not destroy preformed heat-resistant enterotoxins of *S. aureus* and *C. botulinum* B toxin and the emetic toxins (cereulide) of *Bacillus cereus*. Similarly, pasteurization neither destroys the heat-resistant spores of *C. botulinum* nor of *B. cereus*. Therefore, in order to destroy vegetative as well as spores of most pathogens, sterilization treatments are ideal. With the exception of spores of some nonpathogenic thermoresistant bacilli, sterilization can destroy spores of most pathogens including those of *C. botulinum* and *B. cereus*. Additionally, preformed toxins of *S. aureus* and *C. botulinum* in milk and the enterotoxins of *B. cereus* can be destroyed by sterilization techniques [125].

Refrigeration is often not accessible to traditional smallholder milk producers and processors in most rural communities in Africa due to the high initial investment and running costs, and technical problems such as the lack or unreliable supply of electricity. Therefore, boiling or heating raw milk is commonly practiced to improve the safety of milk before consumption by destroying or reducing the growth of pathogenic and spoilage microorganisms. Thus, it is common for milk to be heated several times as a means of preservation before consumption or further processing into other traditional products, a practice that arguably reduces the sensory properties and nutritional value of milk. Also, during the production of African fermented dairy products, raw milk is often boiled or pasteurized for safety and various technological reasons. For example, during the production of nyarmie and fènè, fermented yoghurt-like products in Ghana and Mali, respectively, raw cow milk is heated up to between 65 °C and 80 °C for about 30–50 min [34,127]. Similarly, raw milk may be heated before fermentation occurs in the traditional processing of milk into amabre and mursik in Kenya [128,129], makamo in Uganda [130] and Pendidaam in Cameroon [131,132]. Although the temperature–time combinations used in heating milk in traditional African milk processing are not properly controlled, they can be considered generally as pasteurization, and are therefore capable of completely destroying most spoilage and vegetative human pathogens which may contaminate raw milk [125]. This method however, may be inefficient dependent on the microbial source of contamination or if initial levels of microbial contamination are high, e.g. pasteurization of milk at 72 °C for 15 s was found to be inadequate to render the milk safe due to the high initial counts of bacterial contamination [133].

#### 5.2.2. Natural Fermentation

For the majority of smallholder dairy farmers and milk processors in Africa, fermentation of milk is the cheapest and most convenient method to prolong the shelf-life of milk. Traditional dairy fermentations in Africa are generally spontaneous or completed by back-slopping [34,121]. Thus, these processes do not involve the use of properly defined starter cultures and the fermentation takes place under poorly controlled conditions such as temperature and time. The fermentation process is initiated and carried out by commensal microorganisms present in collection and fermentation containers, the environment or from the hands of processors. Lactic acid bacteria (LAB) and yeasts are predominantly involved in traditional African fermentation of dairy products [34,122,127,134,135,136,137].

Traditional African fermented foods are generally considered to be safe due to the production of antimicrobial compounds by fermenting bacteria and the reduction in pH which contribute to inhibiting the growth of pathogenic microorganisms. The metabolic activities of LAB and yeasts results in a considerable decrease in pH due to production of organic acids (lactic and acetic acids). Additionally, compounds such as diacetyl, hydrogen peroxide and carbon dioxide are produced during LAB and yeasts fermentation [138,139,140]. These organic acids, together with the other compounds, act in synergy as antimicrobials, interfering with various metabolic activities of many pathogenic microorganisms by reducing their internal pH, altering their cell membrane potential and inhibiting active transport, destroying membrane integrity by peroxidation of membranes lipids and denaturing enzymes and DNA [141,142,143]. However, depending on the species and strains of fermenting bacteria, varying amounts of these compounds are produced during milk fermentation. For example, cell-free cultures supernatant of lactic acid bacteria (LAB) strains isolated from nunu, exhibited varying degrees of inhibition against indicator pathogenic strains i.e., *B. cereus* PA24, *S. aureus* ATCC 19095, *E. coli* O157:H7, *L. monocytogenes* ScottA, *Salmonella enterica* Typhimurium ATCC 13311, and *Pseudomonas aeruginosa* BFE 162. Notably, *Lb. fermentum* (10%), *Lb. plantarum* (27%), *Lb. helviticus* (31%), *Leuconostoc mesenteroides* (20%), and *Enterococcus italicus* (5%) exhibited the greatest zones of inhibition [144]. Furthermore, a considerable reduction in counts or complete elimination of Enterobacteriaceae during spontaneous fermentation of milk to produce nunu in rural communities in Ghana has been reported [145].

Other antimicrobial compounds produced during the fermentation of milk such as diacetyl, hydrogen peroxide and bacteriocins have various degrees of antimicrobial activities against potential food spoilage microorganisms. Generally, diacetyl is produced in low concentrations by some LAB as part of their metabolic activities. The antimicrobial activity of diacetyl is enhanced when used synergistically with other antimicrobials such as hydrogen peroxide. Bacteriocins, on the other hand are antimicrobial peptides produced by some bacteria, and typically show activity against closely related species whereas the producer bacteria are unaffected because they possess specific protective mechanisms [146]. Some bacteriocins as nisin has antimicrobial activity against both gram positive and gram-negative bacteria [147], and can inhibit sporulation in bacilli and clostridia [148,149]. Lactic acid bacteria isolated from milk have shown the capacity to produce great diversity of bacteriocins [150]. Bacteriocin-producing cultures can be inoculated in situ as starter or adjunct cultures [35,151], or purified bacteriocins can be added ex situ [152] to improve safety of milk and dairy products. Currently, the commercial application of nisin (Nisaplin, Danisco, Denmark), and pediocin PA1 (MicrogardTM, ALTA 2431, Quest International, USA) as bio-preservative in the dairy industry are accepted. Nisin in synergy with other antimicrobials such as LysH5, the endolysin encoded by staphylococcal bacteriophages, showed strong inhibitory activity towards *S. aureus* Sa9 in-vitro [153]. The viability of strains together with growth conditions e.g. temperature, pH and water activity among others have been found to affect the effectiveness of the use of bacteriocin-producing strains as protective cultures in situ to control pathogens in dairy products [152]. Other LAB bacteriocins such as enterocin AS-48 [154] and lacticin 3147 [155], have shown promising prospects for commercial application as food bio-preservatives. 

Fungal contamination of milk and dairy products can occur along the dairy production chain, posing severe problems, especially in areas where mycotoxin producing moulds frequently occur. However, fermentation by LAB has been reported to be important for the reduction in mycotoxin content. Thus, the antifungal property LAB strains can be exploited in inhibiting fungal growth in various of fermented milk products including yoghurts [156,157,158] and cheeses [159]. Significant reduction in mycotoxin (AFM1) content in contaminated milk was observed as it was processed into yoghurt, cheese and acidified milk [160]. Similar results were observed during the processing of raw milk into Egyptian domaiti cheese and subsequent storage for three months at 20 °C [161]. The reduction in mycotoxin in fermented foods has been attributed to interference of mycotoxin production by LAB during the fermentation process [162]. In addition to the direct interference or production of antifungal compounds to inhibit fungal growth during fermentation, some LAB strains interact with fungal mycotoxins, leading to their inactivation or their removal through cell wall binding [163,164].

#### 5.2.3. Use of Antimicrobial Additives

In order to inhibit the growth of pathogenic and spoilage microorganisms, traditional milk processing in certain parts of Africa employ the use of various medicinal plant parts or their extracts to serve as antimicrobial agents. For example, people of the Maasai community in Kenya add extracts of *Lippia javanica* (stem), *olkingiri* (stem) or *Olea europea* (root) to pasteurized milk before fermenting it into kule naoto. Pretreatment of the fermenting gourd with ashes or charcoal remains of *Olea africana* before filling it with pasteurized milk during the production of kule naoto improves the quality and enhances the safety of the milk product [165]. Microbial counts in plant extract-fermented milk were found to be lower than control, even though pH was higher in the plant extract-fermented milk [166]. During production of mursk, the gourd is somehow sanitized by burning sticks of *Senna didymobotrya* before filling it with pasteurized milk for fermentation [167]. Similarly, the fermentation of camel milk into suusac is preceded by smoking the gourd (fermentation vessel) with *Acacia seyal* [168], a process that is expected to eliminate pathogenic and spoilage microorganisms before the fermentation process takes place in order to ensure safety of the final product.

## 6. Future Perspectives for Improving Food Safety

Milk is highly prone to spoilage; and this challenge is exacerbated by the fact that the warm climate in Africa encourages the growth of both pathogenic and spoilage microorganisms in highly perishable foods like milk. Efforts to improve the microbial safety of milk and dairy products in Africa will require commitment from all key stakeholders in the dairy chain.

### 6.1. Role of Governments and Regulatory Bodies

The role of governments in ensuring the safety of milk and dairy products includes setting the necessary safety standards, performing inspections and putting measures in place to ensure that the standards are met, and having a strong enforcement program. In this context, there is the need for governments in the African continent to set up and legislate laws and regulations on the handling of milk—from farm to cup. Some of the expected standards for milk will include milking under sanitary conditions, cooling milk to refrigeration temperatures (below 4 °C) immediately after milking, transporting milk with vehicles equipped with appropriate cooling facilities, etc. Governments must also incentivise farmers by providing the access to necessary facilities. For example, the provision of centrally placed holding and distribution centres that are equipped with chilling and microbial testing facilities will ensure that milk travels only short distances from time of milking until cooling. This way, milk will not be subjected to significant temperature abuse that promotes spoilage, thus assuring that the milk fed into the food system is safe. There should also be functioning surveillance measures and systems to track and halt the carrying of contaminated milk into the dairy chain, and these surveillance systems must be accessible by the general public. Governments should also perform outbreak investigations to identity sources of contamination in the dairy chain, new pathogens and their food vehicles, as well as gaps in the dairy chain that compromise on food safety. Such investigations can be done in partnership with various academic and research institutions who can use the findings in the training of the next generation of food safety and control staff in the fields of food science, food technology, agriculture and biology. It is worth noting that governments must upgrade the requirements for food safety to support other important requirements such as economic implications (e.g., economic risks that arise from food spoilage), trade partnerships and agreements. Finally, to develop lifelong safe food handling habits among the populace, governments would need to implement a wholistic food safety and hygiene as part of the education curricula beginning with junior and senior high schools to improve food safety cognition among students and promote long-term safe food-handling behaviour.

### 6.2. Role of Dairy Chain Actors

Dairy farmers, distributors and milk processors play the most important role in ensuring the safety of milk and dairy products. The average African dairy farmer must have the knowledge and training in preventive approaches such as hazard analysis and critical control points (HACCP) design and implementation, good hygienic practices (GHPs), and good manufacturing practices (GMPs) in the handling of milk. Knowledge on the use of simple equipment such as mastitis detectors to assess the health of cows, and resazurin kits to estimate microbial loads in milk are also important. Dairy farmers must also have knowledge on good agricultural practices (GAPs) that include good milking practices, humane treatment of cows to promote animal health, ensuring that cows are only fed safe feed and water etc. Farmers will also need to liaise with the necessary governmental bodies to run surveillance systems and ensure an effective recall program in the event that milk is found to be contaminated. Agricultural extension officers can assist with the training of farmers in food quality assurance and management programs. In this regard, farmers and extension officers can peruse training modules and documents produced by FAO, as these documents contain useful information that are feasible for the implementation in resource-poor settings.

Milk distributors and dairy processors also have a role to play in ensuring the safety of milk in the dairy chain. Processing of milk via fermentation into yoghurts, cheeses and other fermented products should be carried out using well defined starter cultures rather than by spontaneous fermentation [169]. The use of starter culture is known to promote consistency in the quality of products [170], shorten fermentation time [171], guarantee product safety [172,173], and allows for the adoption of predictive microbiology principles. Predictive microbiology operates on the assumption that microorganisms will behave (grow and metabolize compounds) in a predictable way under similar environmental conditions (pH, temperature, water activity, etc), making it possible to assess, forecast and quantify microbial populations and their products as a function of environmental conditions [174,175]. 

Moreover, distribution outlets will need to be equipped with the necessary facilities for rapid cooling, pasteurisation, testing for microbial load, and cold transportation of milk. Cold storage and transportation of foods in Africa is a challenge. This is not only because of the warm and humid climate in the continent, but also because many countries in Africa experience erratic power supply [176,177], making it difficult to keep products in cold storage for extended periods of time. This said, there are several innovative technologies and bespoke cooling equipment that can be adopted and used in the dairy sector in Africa. Examples of these include evaporative cooling chambers, also called zero energy cool chambers [178]. However, their value in the dairy chain still needs to be proved. Other innovative technologies that could be useful for ensuring microbial safety in the dairy chain include the development of electricity-independent and solar-powered fridges. Finally, consumers have the responsibility to educate themselves in food safety principles, follow labelling instructions and obey “use by” dates on dairy products. They must also keep up to date with dairy surveillance systems published by government and food processors and demand safe and quality milk and dairy products from suppliers.

## 7. Conclusions

The consumption of unpasteurized milk and dairy products continue to pose significant health risk to consumers due to the persistence of microbial pathogens of significant safety concern in the dairy chain in Africa. Notwithstanding, the production and consumption of milk in Africa is projected to grow in the next few decades. While this forecast has positive economic and nutritional prospects for the continent, it also raises serious concerns for public health, considering that milk and dairy products in Africa are frequently contaminated with pathogenic microorganisms. Therefore, there is the need to deliberately put in place systems to ensure the safety of milk and dairy products in the African continent to avert any future detrimental health and economic impact. To minimize the health risks associated with the consumption of milk and dairy products, all food-chain operators, including dairy farmers, processors, distributors, retailers and consumers would need to take the required steps to maintain food-safety. Governments and regulatory agencies have a crucial role to play in engaging with all stakeholders to establish national and regional controls and standards, including inspection and surveillance to ensure effective controls and conditions for safe production, transportation and storage of milk and dairy products. The establishment and implementation of control measures and standards should also take into account the unique characteristic of the dairy chain in the particular African country. Recognition of smallholder dairying and informal markets through training and certification would be very significant in ensuring the safety of milk and dairy products. The role of education in ensuring production, distribution and processing of safe milk cannot be overemphasized. If consumers are well-informed about the dangers of consuming contaminated milk, they will demand the highest safety standards from the milk distributors and regulatory authorities. The outcome of all these would be a well-functioning dairy system that not only brings economic incentives, but also protects the health of the consumers.

## Figures and Tables

**Table 1:** Main categories of food safety hazards associated with milk and dairy products (adopted from the Food and Agricultural Organization (FAO), [13]).

Biological Hazards	Chemical Hazards	Physical Hazards
Pathogenic bacteria (including toxins produced by bacteria) Toxigenic moulds/fungi Parasites Viruses Other biological hazards	Naturally occurring toxins Direct and indirect food additives Pesticide residues Veterinary drug residues Heavy metals Environmental contaminants Chemicals from packaging material	Metal fragments Bone fragments Glass pieces Insects or their parts Jewellery Stones/soil/dust Hair/fur

**Table 2:** Major microbiological risk factors and their implications for safety in the dairy chain in Africa.

Step in Dairy Chain	Important Risk Factors	Implications for Milk Safety
Primary production	Diseases (mastitis) Housing, bedding and husbandry Feed and water quality Waste management	Increased shedding of pathogens directly into milk from diseased animals (including asymptomatic carriers). Poor housing and husbandry practices increase the risk of udder contamination due to high stocking, concentration of waste, stress and soiled bedding, leading to contamination of milking environment and raw milk. Increased risk of milk contamination can result from using poor quality water for stock drinking, teat washing and cleaning. Contaminated or poorly prepared feed may increase faecal shedding of pathogens into milk and milking environment.
Milk collection	Milking practices Equipment cleaning Personnel hygiene	Poor milking practices, including dirty, chapped or cracked teats, insufficient cleaning and maintenance of milking equipment, and poor personnel hygiene can lead to direct contamination of raw milk with pathogens.
Raw milk storage	Availability and efficiency of cold storage facilities	Inappropriate temperature control of raw milk, coupled with the usually high temperature in the region and erratic power supply, can lead to accelerated growth of pathogens in milk during storage.
Packaging	Packaging Equipment and material	Poor packaging, inappropriate packaging materials and poor hygiene can contribute to cross contamination of milk or open up milk to contamination from the environment.
Transportation and distribution	Transportation mode Road network between milk collection centres and market centres Maintenance of cold chain	Transporting of raw milk between farms and market centres by foot, bicycles, motorbikes or other means without a proper cold chain enables growth of pathogens. Poor road network systems increase the time for transportation and distribution of raw milk, and coupled with poor cold chain facilities, allows the rapid growth of pathogens in raw milk.
Traditional milk processing	Pasteurization/thermal treatment Fermentation practices Personnel hygiene and sanitation of processing environment.	Inadequate pasteurization temperatures may not be able to eliminate pathogens in already contaminated milk, and may even encourage the faster growth of pathogens. Spontaneous fermentations (without properly defined starter cultures), coupled with poor time/temperature controls can expose fermented products to pathogenic microorganisms. Poor sanitation of processing environments and personal hygiene by milk processors can lead to a direct contamination of processed milk products with pathogenic microorganisms.
Consumer practices	Storage temperature at home storage Adherence to handling instructions and good personal hygiene	Poor refrigeration during home storage of both raw and processed milk can accelerate the proliferation of pathogenic microorganisms. Lack of proper hygiene and nonadherence to handling instructions can lead to contamination and proliferation of pathogenic microorganisms.
